# Survival Motor Neuron Protein is Released from Cells in Exosomes: A Potential Biomarker for Spinal Muscular Atrophy

**DOI:** 10.1038/s41598-017-14313-z

**Published:** 2017-10-24

**Authors:** Leslie A. Nash, Emily R. McFall, Amanda M. Perozzo, Maddison Turner, Kathy L. Poulin, Yves De Repentigny, Joseph K. Burns, Hugh J. McMillan, Jodi Warman Chardon, Dylan Burger, Rashmi Kothary, Robin J. Parks

**Affiliations:** 1 Regenerative Medicine Program, Ottawa Hospital Research Institute, Ottawa, Ontario Canada; 2 Department of Biochemistry, Microbiology and Immunology, University of Ottawa, Ottawa, Ontario Canada; 3 University of Ottawa Centre for Neuromuscular Disease, Ottawa, Ontario Canada; 4 Kidney Research Centre, Ottawa Hospital Research Institute, Ottawa, Ontario Canada; 5 Department of Cellular and Molecular Medicine, University of Ottawa, Ottawa, Ontario Canada; 6 Department of Pediatrics, Children’s Hospital of Eastern Ontario, University of Ottawa, Ottawa, Canada; 7 Children’s Hospital of Eastern Ontario Research Institute, Ottawa, Ontario Canada; 8 Division of Neurogenetics, Department of Genetics, Children’s Hospital of Eastern Ontario, Ottawa, Canada; 9 Department of Medicine, University of Ottawa, Ottawa, Ontario Canada

## Abstract

Spinal muscular atrophy (SMA) is caused by homozygous mutation of the survival motor neuron 1 (*SMN1*) gene. Disease severity inversely correlates to the amount of SMN protein produced from the homologous *SMN2* gene. We show that SMN protein is naturally released in exosomes from all cell types examined. Fibroblasts from patients or a mouse model of SMA released exosomes containing reduced levels of SMN protein relative to normal controls. Cells overexpressing SMN protein released exosomes with dramatically elevated levels of SMN protein. We observed enhanced quantities of exosomes in the medium from SMN-depleted cells, and in serum from a mouse model of SMA and a patient with Type 3 SMA, suggesting that SMN-depletion causes a deregulation of exosome release or uptake. The quantity of SMN protein contained in the serum-derived exosomes correlated with the genotype of the animal, with progressively less protein in carrier and affected animals compared to wildtype mice. SMN protein was easily detectable in exosomes isolated from human serum, with a reduction in the amount of SMN protein in exosomes from a patient with Type 3 SMA compared to a normal control. Our results suggest that exosome-derived SMN protein may serve as an effective biomarker for SMA.

## Introduction

With an occurrence of 1 in 10,000 live births and a carrier frequency of 1 in 40, spinal muscular atrophy (SMA) is the most common cause of death by a genetic disease in newborns^1,2^. This autosomal recessive disorder is characterized by the degeneration of α-motor neurons, resulting in progressive atrophy of skeletal muscle^3,4^. However, recent studies have shown that many different cell types and tissues also show impaired function in SMA^5–8^. SMA is caused by a deficiency in full length survival motor neuron (SMN) protein, due to homozygous deletion or mutation of the *SMN1* gene^9,10^. Complete loss of SMN protein results in embryonic lethality in mice^11^, and likely also in humans^12,13^. However, humans have a second, highly homologous copy of the *SMN1* gene, termed *SMN2*. Although these two genes share greater than 99% nucleotide identity^14^, a synonymous point mutation in the *SMN2* gene affects splicing of the pre-mRNA resulting in production of predominantly an mRNA lacking exon 7^15^. The resulting SMNΔ7 protein is relatively unstable and cannot perform all of the functions of the full length SMN protein^16–18^. Approximately 10% of the transcripts from the *SMN2* gene retain exon 7 and produce full length SMN protein^15^. Since the *SMN* locus is variably amplified in humans, the number of copies of the *SMN2* gene that a patient has can significantly influence the severity of the disease^19–21^, as each additional copy of the *SMN2* gene brings a patient closer to wildtype levels of SMN protein^22^. While SMN protein has been implicated in intracellular processes including splicing^23,24^, translational regulation^25^, R-loop resolution^26^, intracellular transport^27–29^ and actin dynamics^30,31^, the exact cause of SMA pathogenesis is currently unclear.

Very recently, an antisense oligonucleotide (ASO)-based therapy, known as Nusinersen, or by its market name Spinraza, has been approved by the Unites States Food and Drug Administration (FDA), Health Canada and the European Union for treatment of SMA^32,33^. Nusinersen blocks an intronic splicing silencer in intron 7, thus promoting the inclusion of exon 7 in the *SMN2*-derived mRNA, resulting in greater production of full length SMN protein^34,35^. Recent results with gene therapy approaches to disease correction have also shown great promise in human clinical trials^36^, while several other therapies have shown encouraging results in preclinical and clinical studies^37–39^. However, currently available clinical outcome measures often lack sensitivity to identify rapid responses to therapies, thereby necessitating long clinical trials particularly in patients with SMA type 2 or 3. Furthermore, clinical strength testing by MRC grading, 6 minute walk test or grip strength are not feasible for younger or more severely affected patients. Common motor outcome measurement tests in infants and children with SMA include Children’s Hospital of Philadelphia Test of Neuromuscular Disorders (CHOP-INTEND), Hammersmith Functional Motor Scale (HFMS), HFMS-Expanded or Hammersmith Infant Neurological Examination (HINE)^40^. However, many of these tests can be limited by subjective assessment of the evaluator and intra-rater reliability. A quantitative, biological component would be an ideal complimentary assay.

One of the easiest and least-invasive methods to provide insight into disease severity and prognosis would be to identify a blood-based biomarker that accurately reflect the SMA disease state. To this end, several researchers have analyzed peripheral blood mononuclear cells (PBMCs) from patients with SMA for a variety of disease markers, including total SMN transcript level, relative full-length *versus* SMNΔ7 mRNA transcript level, and protein level^41–45^. Although these studies demonstrated a trend between motor function and changes in several markers, no statistical correlation was observed. Following treatment of a mouse model of SMA with therapeutic ASO, Arnold *et al*.^46^ did observe significant changes in several plasma-derived biomarkers, suggesting that these may be of some value in monitoring therapeutic efficacy in patients. However, additional accurate and sensitive biomarkers are desired that more closely correlate with clinical phenotype or disease presentation in patients with SMA.

Cells produce a variety of extracellular vesicles, including exosomes, microparticles, and apoptotic bodies^47^. Exosomes are nano-sized particles that are approximately 30–100 nm in diameter and are involved in intercellular communication. Exosomes contain a heterogeneous mix of protein and nucleic acid which are reflective of the cell from which they were derived^48^. It is this unique feature that has prompted many research groups to examine whether exosomes can act as effective biomarkers for a diverse set of diseases, from cancer to neurological disorders^49–52^. In this study, we evaluated the SMN protein content of exosomes isolated from tissue culture and animal models of SMA, and performed an analysis regarding whether exosome-derived SMN protein may be an effective biomarker for SMA.

## Results

### SMN protein is released from cells into the surrounding milieu in culture

**Figure 1:**
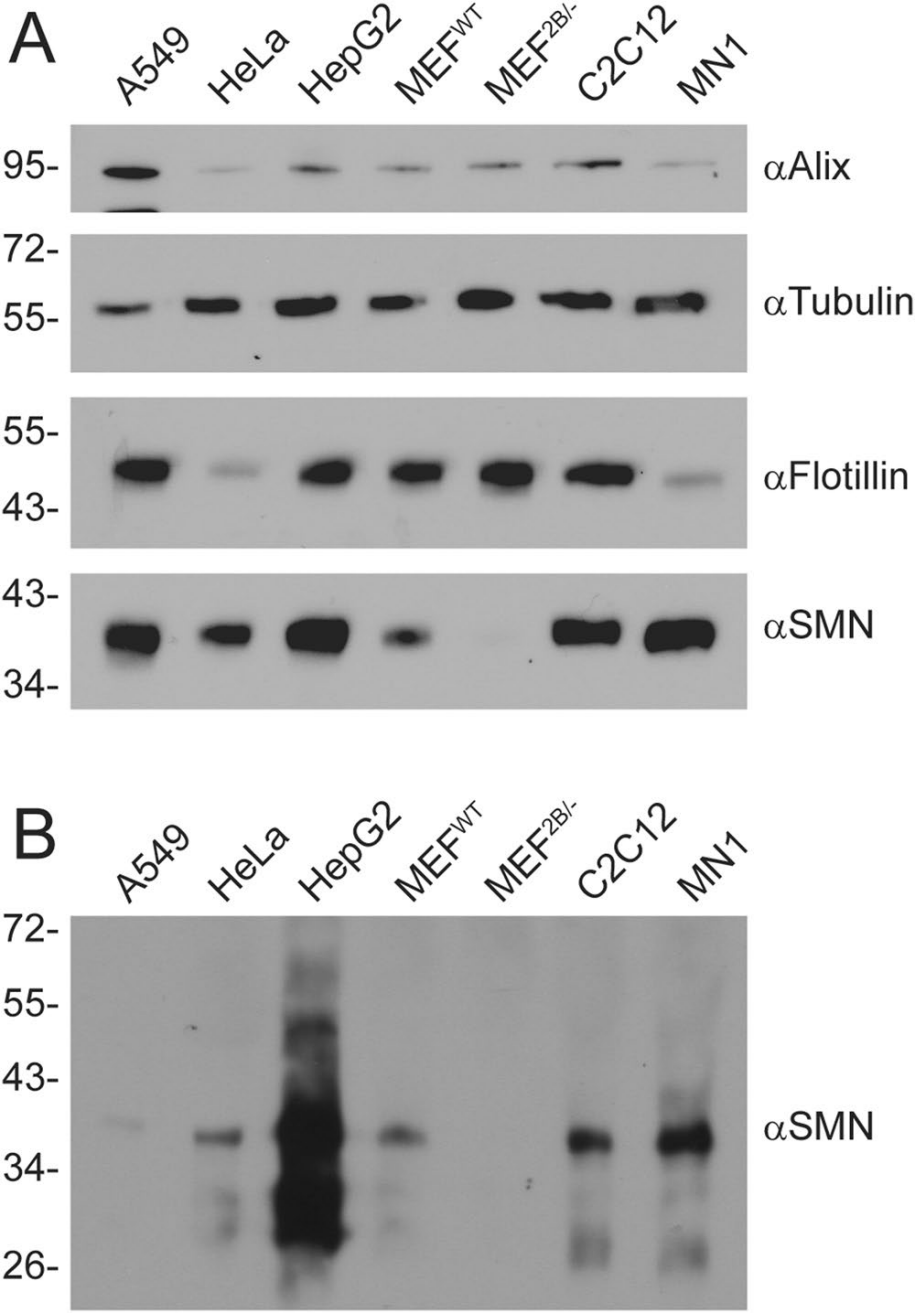
SMN protein is released from cells into the extracellular milieu. Various cell lines were plated in 35 mm dishes for 24 h. Media was removed, the cells lysed with protein loading buffer to analyse intracellular protein content (Panel A), and the media subjected to TCA precipitation to analyse extracellular protein content (Panel B). Equal volumes of protein sample were subjected to SDS-PAGE, transferred to a nylon membrane and probed by immunoblot with antibodies to Alix, tubulin, flotillin, or SMN. Data is representative of *n* = 3.

As an initial test to determine if SMN protein was found extracellularly, we examined protein contained in TCA precipitates from a variety of cell lines. As shown in Fig. 1A, all cell lines expressed varying levels of SMN protein within the cells. As expected, total cell lysates prepared from MEFs derived from the *Smn* ^*2B*/−^ mouse, an intermediate model of SMA^53^, showed barely-detectable levels of SMN protein. TCA precipitates of media from these cell lines revealed that SMN protein was indeed found in the extracellular milieu (Fig. 1B). As expected, medium from MEF^2B/−^ cells did not contain detectable levels of SMN protein. In short, SMN protein can be found extracellularly when expressed at significant levels in the host cell.

### SMN protein is contained in extracellular vesicles derived from cells in culture

**Figure 2:**
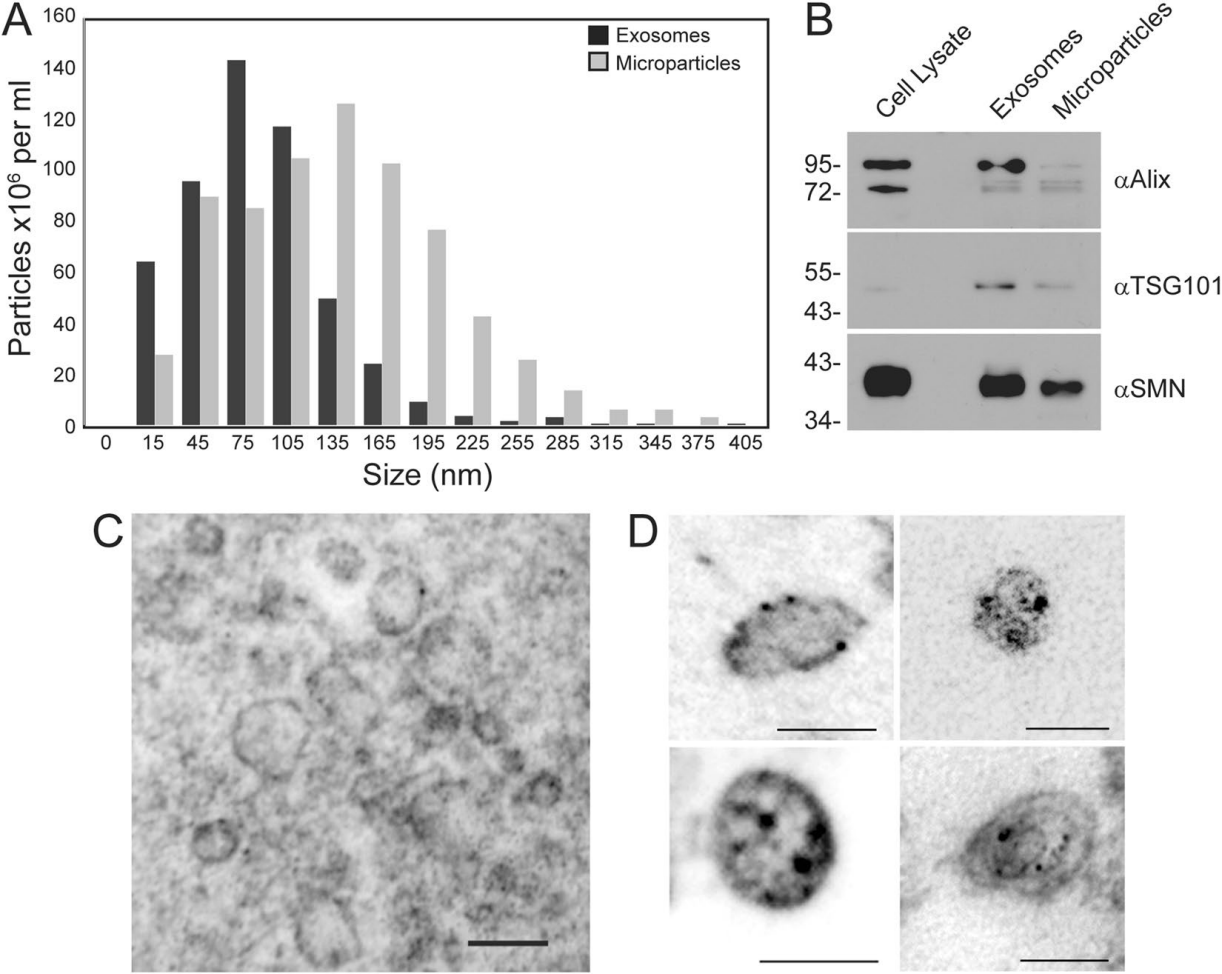
SMN protein is released from cells inside of extracellular vesicles. Panel A: Exosomes and microparticles were isolated from A549 cells, grown in medium supplemented with vesicle-depleted FBS, using differential centrifugation and their size profiles were determined using nanoparticle tracking analysis. Panel B: Protein from extracellular vesicles (5 μg) was subjected to SDS-PAGE and the resulting immunoblot was probed for Alix, TSG101, and SMN. Data is representative of *n* = 2. Panel C: Immunogold labeling of SMN protein was used to demonstrate the presence of SMN protein within A549-derived exosomes, scale bar represents 100 nm. Panel D: Higher magnification of electron microscopy images showing SMN protein contained in exosomes, scale bar represents 100 nm. Four representative images are shown.

SMN protein does not have an N-terminal secretory peptide, and would not be expected to be naturally secreted from cells. Cells release a variety of extracellular vesicles, including microparticles (0.1–1 µm) and exosomes (30–100 nm)^47^. These vesicles contain a mix of protein and nucleic acid characteristic of the host cell. To determine if SMN protein was released from cells in extracellular vesicles, we isolated microparticles and exosomes through differential centrifugation of medium from A549 cells, and subjected the samples to immunoblot for SMN protein. As shown in Fig. 2A, the size distribution of particles obtained from our differential centrifugation protocol were consistent with the expected sizes for both the microparticle and exosome fractions. SMN protein was associated with both types of particles, as were the exosome and microparticle proteins Alix and TSG101 (Fig. 2B). To confirm that the SMN protein was indeed contained within the exosomes, and not simply co-purifying with the particles, we performed electron microscopy using immunogold labeling to determine the localization of SMN protein within the particles. Extracellular vesicles of varying size were evident, some of which were labeled with the gold-tagged antibody (Fig. 2C). We did not detect significant labeling outside of vesicles, suggesting that all the SMN protein in the extracellular vesicle purifications was contained within the vesicles, and not the result of co-purification of free protein. At higher magnification (Fig. 2D), we clearly detected SMN protein within exosomes and microparticles. Thus, SMN protein is naturally released from cells in extracellular vesicles.

### The quantity of SMN protein within exosomes correlates to the level expressed within the host cell

**Figure 3:**
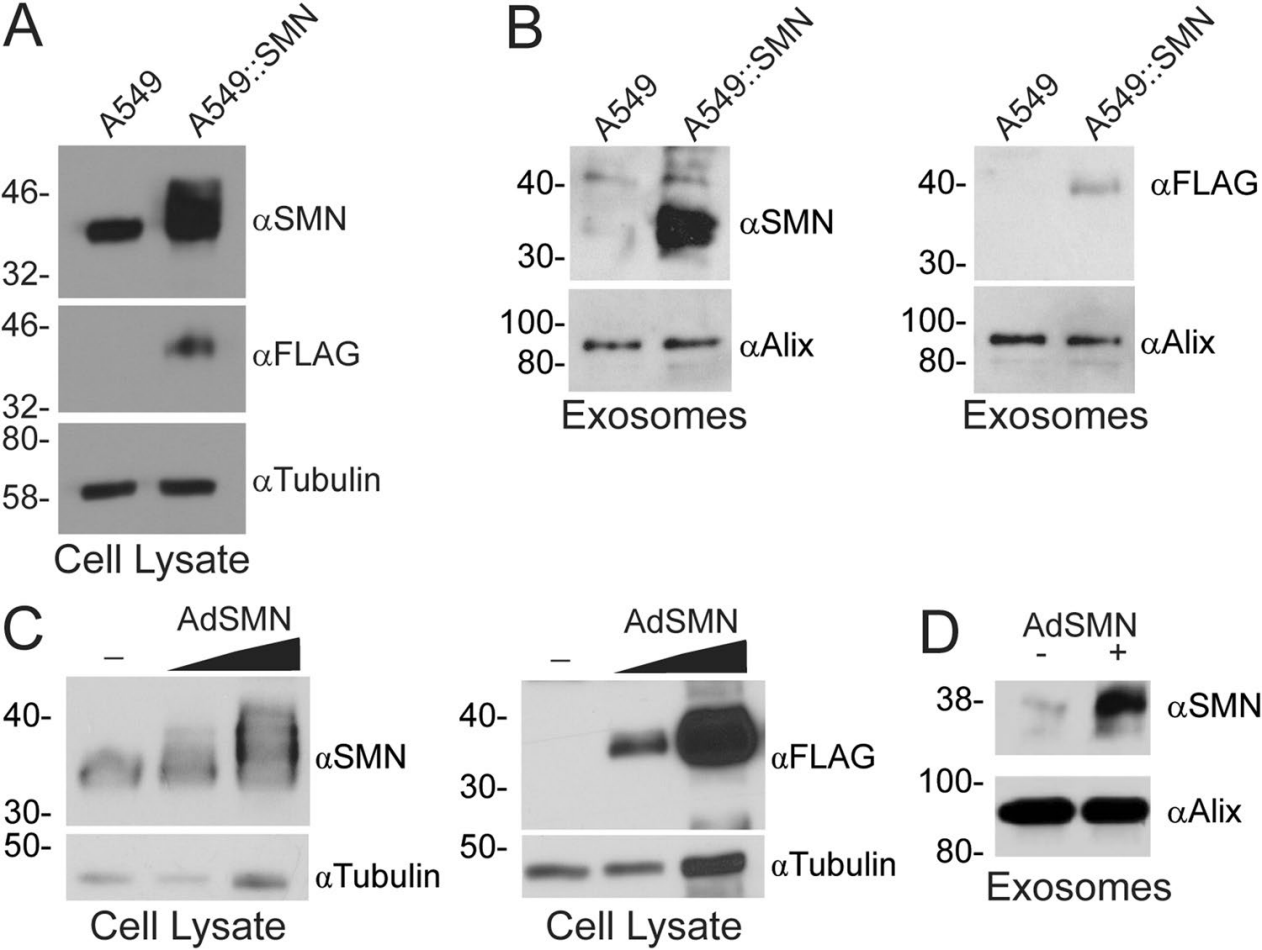
The quantity of SMN protein within exosomes reflects the level expressed within the cell from which they are derived. Panel A: A stable A549-based cell line that overexpresses a FLAG-tagged version of SMN protein was generated, designated A549::SMN cells, and the quantity of intracellular SMN protein was analysed by immunoblot using antibody to SMN and FLAG, relative to the parental A549 cell line. Equal protein loading was confirmed by probing the membrane with tubulin. Data is representative of *n* = 3. Panel B: Exosomes were isolated from medium of A549 or A549::SMN cells, 3 µg of the resulting samples were separated by SDS-PAGE on duplicate blots, and the resulting immunoblots were probed for SMN and Alix (loading control), or FLAG and Alix. Data is representative of *n* = 3. Panel C: A549 cells were infected at an MOI of 10 or 100 with AdSMN (or mock infected) and, 24 h post-infection, crude cellular protein lysates were prepared and analysed by immunoblot on duplicate blots for SMN and tubulin (loading control), or FLAG and tubulin. Data is representative of *n* = 3. Panel D: A549 cells were infected with an MOI 50 and media was collected for exosome purification. Equal volume of each sample was separated by SDS-PAGE and analysed by immunoblot for SMN and Alix (loading control). Data is representative of *n* = 3.

Research on exosomes represents a novel, emerging field in science due to the recent discovery of their involvement in RNA and protein transport between cells^47,54^, including cells of the nervous system^55^. Proteins contained in exosomes often reflect the state of the host cell, and thus exosomes are being developed as biomarkers for a variety of disease states^56–58^. We therefore examined whether the quantity of SMN protein contained in exosomes correlated with the level expressed within the host cell using two different approaches. First, we developed an A549-based cell line that overexpressed a FLAG-tagged version of SMN protein, designated A549::SMN cells. The presence of the FLAG-tag causes a shift in the size of the protein, which appears as a doublet or an expanded area of reactivity on immunoblot with antibody to SMN protein (Fig. 3A). Exosomes isolated from the media of the parental A549 and A549::SMN cell lines showed that significantly higher levels of SMN protein were found in exosomes from the over-expressing cell line, and FLAG-immunoreactivity was only detected in the exosomes derived from the FLAG-SMN-expressing cell line (Fig. 3B). In a second experiment, A549 cells were infected with increasing quantities of AdSMN, which also expresses a FLAG-tagged SMN protein, and the intracellular and exosome-derived levels of SMN protein were examined 24 h later. As shown in Fig. 3C, increasing the amount of AdSMN applied to the cells was accompanied by an increased quantity of SMN protein within cells, as detected by both anti-SMN and anti-FLAG antibody. Exosomes isolated from AdSMN-infected cells showed a dramatic increase in the quantity of SMN protein relative to exosomes isolated from untreated cells (Fig. 3D). Thus, the quantity of SMN protein contained within exosomes is reflective of the level within the host cell, at least for cells overexpressing the protein.

### The quantity of SMN protein within exosomes correlates to the level expressed within the host cell in tissue culture models of SMA

**Figure 4:**
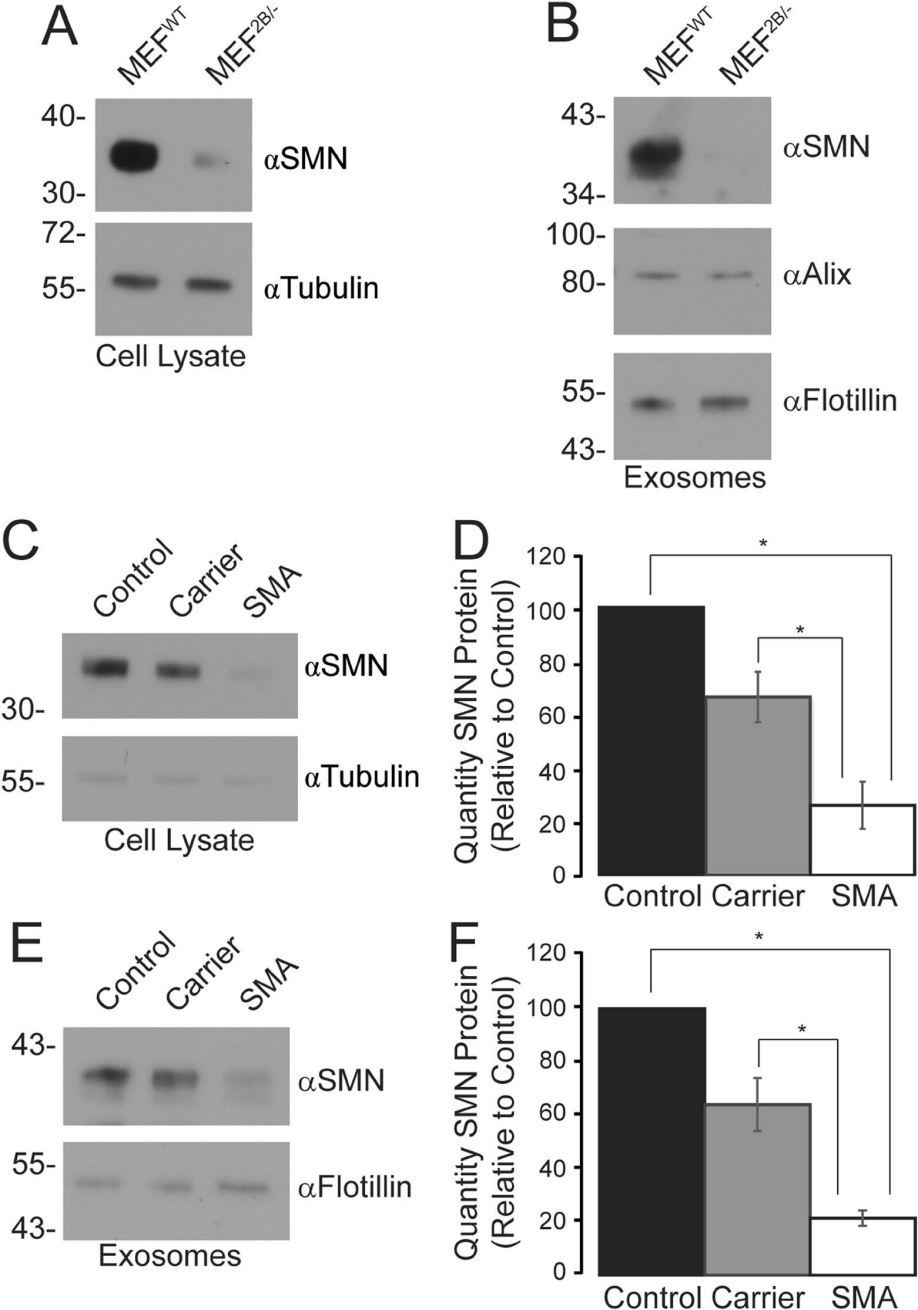
The quantity of SMN protein in exosomes reflects the intracellular levels in fibroblasts derived from a mouse model of SMA and patients with type I SMA. Panel A: Crude protein cell lysates (5 µg) from MEFs derived from wildtype mice (MEF^WT^) or *Smn* ^*2B*/−^ mice (MEF^2B/−^), a mouse model of SMA, were analysed by immunoblot for SMN and tubulin (loading control). Data is representative of *n* = 3. Panel B: Exosomes were isolated from the medium of MEF^WT^ and MEF^2B/−^, 3 µg of the resulting samples were separated by SDS-PAGE and analysed by immunoblot for SMN, Alix, and flotillin (loading control). Data is representative of *n* = 3. Panel C: Crude protein cell lysates (5 µg) from normal, SMA carrier or SMA type I human fibroblasts were analysed by immunoblot for SMN and tubulin (loading control). Data is representative of *n* = 3, and is quantified in Panel D. Asterisks (*) indicates significant differences between groups, determined by Bonferroni post-hoc analysis (*p* < 0.05). Panel E: Exosomes were isolated from the medium of normal, carrier or SMA type I fibroblasts, 3 µg of the resulting samples were separated by SDS-PAGE and analysed by immunoblot for SMN, Alix, and flotillin (loading controls). Data is representative of *n* = 3, and is quantified in Panel F. Asterisks (*) indicates significant differences between groups, determined by Bonferroni post-hoc analysis (*p* < 0.05).

We next examined two tissue culture models of SMA to determine if the quantity of SMN protein contained in exosomes also reflected that of the host cell. An examination of the intracellular levels of SMN protein in MEFs derived from the *Smn* ^*2B*/−^ mouse model of SMA or wildtype mice showed that, as expected, there are significantly reduced levels of SMN protein in the SMA model (Fig. 4A). Exosomes isolated from medium from these two cell lines also showed a significant reduction in the level of SMN protein contained within the exosomes released from MEF^2B/−^ cells (Fig. 4B), below the level of detection for this assay.

We next examined the intracellular and exosome-derived quantity of SMN protein in fibroblasts derived from a human patient with SMA or a carrier, relative to a fibroblast cell line from a normal control. As expected, the intracellular quantities of SMN protein within fibroblasts derived from carrier or type I SMA patients was considerably less than in fibroblasts derived from the normal control (Fig. 4C). Indeed, the quantity of intracellular SMN protein in the fibroblasts from SMA patients was statistically significantly lower than both the control and carrier fibroblasts (Fig. 4D). Exosomes isolated from the medium from these three cell lines showed a similar trend (Fig. 4E), and a statistically significant difference was determined across the three groups (Fig. 4F). Regression analysis showed that there was a high correlation between the genotype of the cell fibroblast cell line (control, carrier, SMA) and SMN protein levels (R^2^ = 0.922, *p* < 0.001). Thus, the levels of SMN protein within exosomes reflects the disease or carrier state, at least in tissue culture models of SMA.

### Cell culture models of SMA showed elevated concentrations of exosomes in the medium

**Figure 5:**
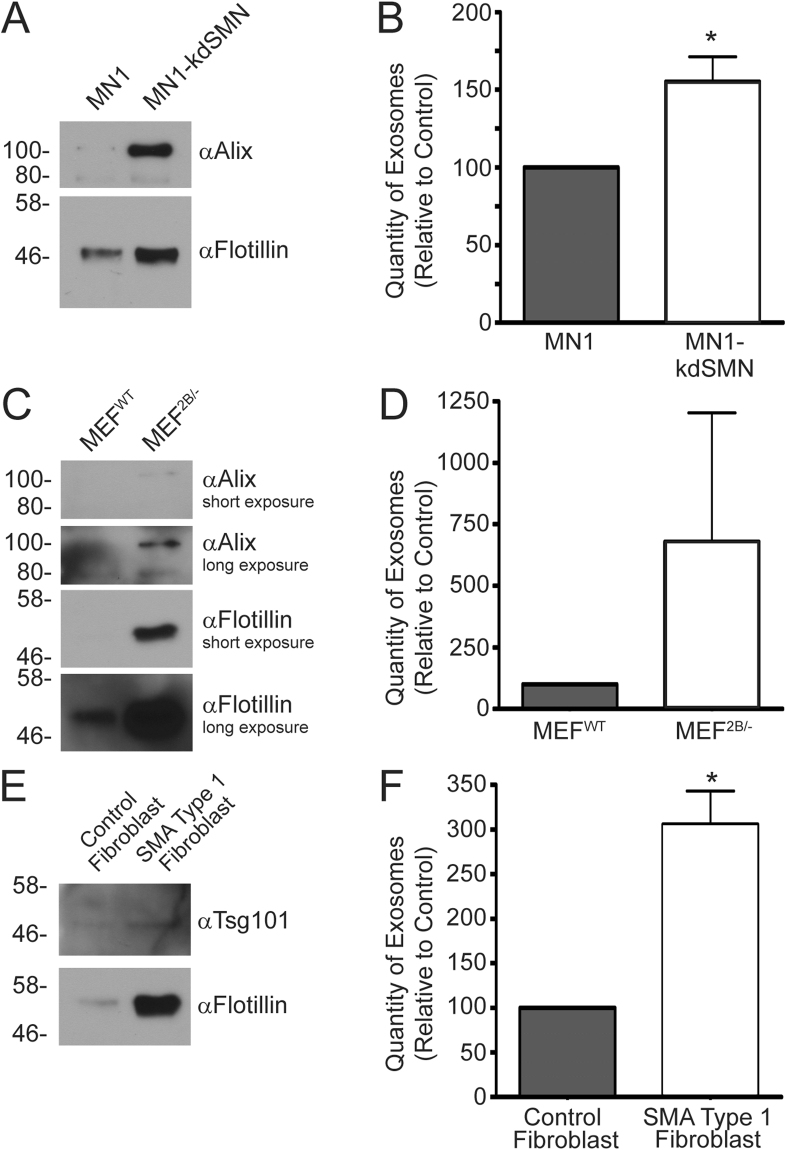
Tissue culture models of SMA show enhanced levels of exosomes in the medium. Exosomes were isolated from control (MN1, MEF^WT^, normal control human fibroblasts) and SMN protein-depleted cell culture models (MN1-kdSMN, MEF^2B/−^ and SMA type I fibroblasts) using Exoquick. Panels A, C and E: Equivalent volumes of the isolated exosomes were separated by SDS-PAGE and analyzed by immunoblot for SMN, or exosome markers Alix, flotillin, or Tsg101. Panels B, D, and F: The concentration of exosomes in the samples was determined by nanoparticle tracking analysis. Data is representative of *n* = 3–4, and is presented as average particle concentration and standard deviation, normalized to the control for each cell type. An asterisk (*) indicates p < 0.05. Statistical analysis was performed using an unpaired Student’s t-test. Exosomes were isolated from cells grown in medium supplemented with microvesicle-depleted FBS.

During our studies designed to evaluate SMN protein content in exosomes, we noted enhanced recovery of exosomes from the cell culture models of SMA relative to control cells, suggesting there was a greater accumulation of exosomes in the medium. To examine this phenomenon more closely, we used two approaches. First, we qualitatively compared by immunoblot exosome protein marker intensity after separating equal volumes of exosome sample isolated from medium from several models of SMA relative to their respective controls. Second, we used nanoparticle tracking analysis to determine the concentration of exosomes isolated from the two types of cells. For the MN1 motor neuron-like cells, analysis of equal volumes of isolated exosomes from culture medium showed that both Alix and flotillin exosomal markers were elevated in the MN1-kdSMN cell line that expresses reduced levels of SMN protein compared to the parental cell line (Fig. 5A). Quantification of the concentration of exosomes in the medium samples showed that the MN1-kdSMN cell line contained almost 60% more exosomes, which was statistically significant (p < 0.05) (Fig. 5B). A similar trend was noted for MEF^2B/−^ compared to MEF^WT^ fibroblasts: MEF^2B/−^ cells showed elevated signal intensity for the Alix and flotillin exosome markers by immunoblot, suggesting a higher concentration of exosomes (Fig. 5C). Although this trend was also observed when the concentration of exosomes was determined by nanoparticle tracking analysis, it was not statistically significant (Fig. 5D). Finally, exosomes isolated from the medium from fibroblasts from a patient with type I SMA showed significantly elevated signal intensity for exosomal markers Tsg101 and flotillin compared to fibroblasts (Alix was poorly detectable in these samples, data not shown) (Fig. 5E). The elevated amount of exosomes isolated from the medium from type I SMA fibroblast, relative to normal control fibroblasts, was confirmed by nanoparticle tracking analysis as almost a 3-fold enhancement in exosome concentration, which was statistically significant (p < 0.05) (Fig. 5F). Of note, cells depleted of SMN protein can show a reduced growth rate^59,60^, and therefore a reduced number of cells may be present in the cultures depleted of SMN protein relative to their respective controls. Thus, the concentration of exosomes present in the medium from SMN-depleted cells shown in Fig. 5 may be an underestimate. Treatment of type I SMA fibroblasts with an adenovirus vector expressing the SMN protein, thus restoring SMN protein levels with the cell, resulted in a trend toward normalization of exosomes release (Supplementary Figure S1). Taken together, these results suggest that cells with reduced levels of SMN protein show enhanced accumulation of exosomes.

### SMN protein levels in exosomes isolated from serum from a mouse model of SMA reflect the disease state

**Figure 6:**
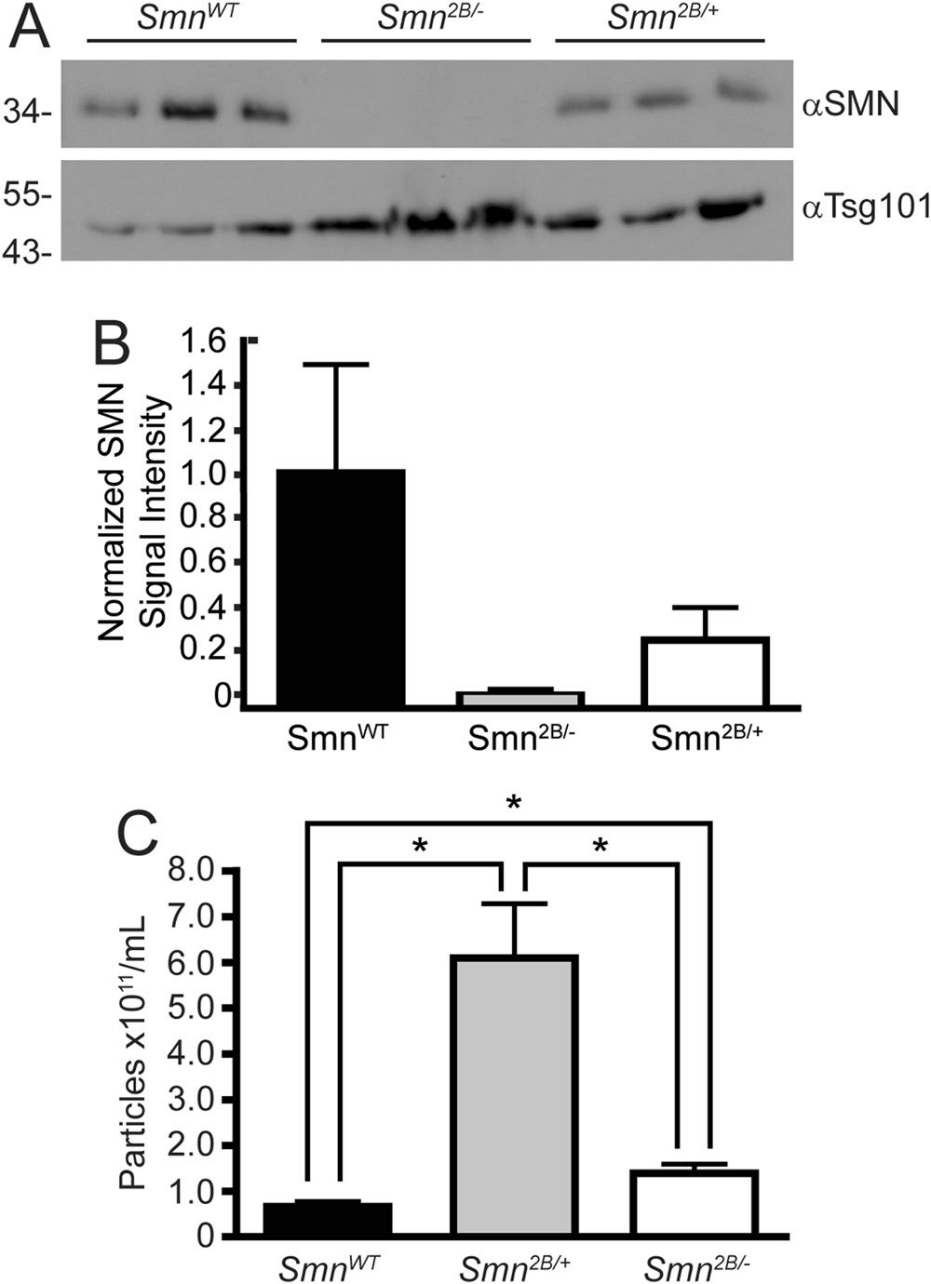
A mouse model of SMA shows enhanced levels of exosomes in serum, which contain a reduced quantity of SMN protein relative to wildtype mice. Panel A: Exosomes were isolated from control, *Smn* ^*2B/+*^ (carrier) and *Smn* ^*2B*/−^ (affected) mouse serum of a C57Bl/6 background using Exoquick. Equivalent volumes of exosomes were separated by SDS-PAGE and analysed by immunoblot for SMN and Tsg101 (loading control). Panel B: Densitometry analysis of the image in Panel A. The average normalized signal intensity is shown, and the error bars represent the range in signal intensity. Panel C: Exosome particle concentration was determined for the serum-derived exosomes from control, carrier and affected mice. Data is representative of *n* = 6, and is presented as average particle concentration and standard deviation. An asterisk (*) indicates p < 0.05. Statistical analysis was performed using a One-Way ANOVA followed by a Games-Howell t-test.

A useful biomarker of disease should not only be sensitive but also be able to be monitored using minimally invasive techniques. Attempts to develop a blood-based screen for SMA, using protein or RNA from PBMCs, have not been successful^41–44^. We asked whether exosomes isolated from serum from a mouse model of SMA reflected the disease state of the animal. We used a commercially available kit to isolate exosomes from the serum of three mice each of control, carrier *Smn* ^*2B*/*+*^, and affected *Smn* ^*2B*/−^, all on a C57Bl/6 background. As shown in Fig. 6A and B, the quantity of SMN protein contained in exosomes did indeed reflect the disease state of the mice – wildtype mice had high levels of SMN protein, heterozygous *Smn* ^*2B/+*^ mice had intermediate levels of protein, and homozygous *Smn* ^*2B*/−^ had low or undetectable levels of SMN protein. Consistent with our results with the tissue culture models of SMA, we also observed a greater recovery of exosomes in samples isolated from the mouse model of SMA relative to control animals (p < 0.05) (Fig. 6C). Interestingly, we also observed elevated levels of circulating exosomes in heterozygous *Smn* ^*2B/+*^ mice, which was statistically significantly different from both affected and control mice. Taken together, these data suggest that analysis of SMN protein levels in serum-derived exosomes, or perhaps relative concentration of exosomes in serum, may represent a new biomarker for SMA.

### SMN protein is detected in human serum-derived exosomes

**Figure 7:**
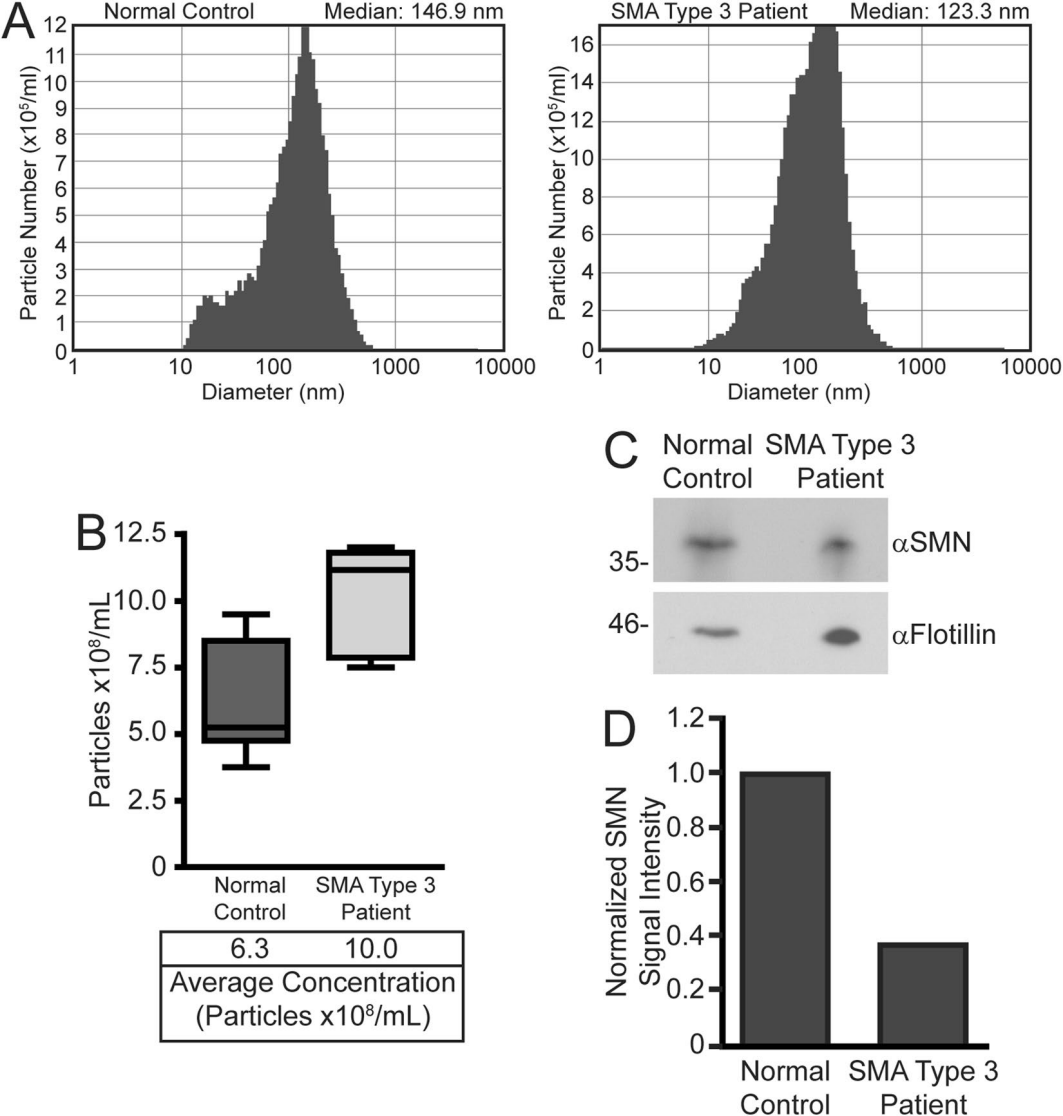
A patient with Type 3 SMA has an elevated level of exosomes in serum, which contained a reduced quantity of SMN protein relative to a normal control. Panel A: Particle diameter of human serum-derived exosomes isolated using Exoquick reagent was determined using nanoparticle tracking analysis for both a normal control (left panel) and a patient with Type 3 SMA (right panel). Panel B: The concentration of exosomes in serum samples from a patient with Type 3 SMA was compared to normal control human serum. The Zetaview ParticleMetrix system quantifies vesicles at 11 camera positions, which is represented in the boxplot. Panel C: Equal volume of serum-derived exosome samples from a patient with type 3 SMA or normal control were separated by SDS-PAGE and subjected to immunoblot analysis for exosomal marker flotillin (loading control) or SMA. Panel D: Densitometry analysis of the image in Panel C. SMN signal intensity is normalized to that of the flotillin loading control.

Lastly, we examined whether SMN protein could be detected in exosomes isolated from human serum. Our protocol for isolating human serum-derived exosomes using Exoquick reagent yielded particles within the appropriate size range (Fig. 7A), and which were of a size consistent with other studies^61^. Quantification of exosomes within the serum samples using nanoparticle tracking analysis showed that, as we observed in cells in culture and in a mouse model of SMA, there was a higher level of circulating exosomes in the serum sample from the patient with Type 3 SMA relative to the normal control (Fig. 7B). Equal volumes of the serum-derived exosomes were next analyzed by immunoblot for the exosomal marker flotillin or SMN protein. Consistent with the higher concentration of exosomes in the serum sample from the Type 3 SMA patient, we observed a higher signal intensity for flotillin in the sample from the patient from SMA relative to the normal control (Fig. 7C). When the SMN protein immunoblot signal was normalized to the loading control (flotillin), we detected a ~60% reduction in the relative amount of SMN protein contained in the serum-derived exosome sample from the patient with Type 3 SMA compared to normal control (Fig. 7D). Taken together, these results suggest that analysis of the quantity of SMN protein isolated from serum-derived exosomes, or the concentration of serum-derived exosomes itself, may be a new method to monitor SMA disease or response to therapy in human patients.

## Discussion

SMA is a debilitating neuromuscular disorder caused by reduced levels of full-length SMN protein. Approval of nusinersen, an ASO designed to promote retention of exon 7 in the mRNA transcript derived from the *SMN2* gene, by the Food and Drug Administration in the USA has provided the first effective therapy to treat SMA^32,33^. Several other approaches for the treatment of SMA are currently undergoing clinical testing, including the use of gene therapy to deliver a “good” copy of the *SMN1* cDNA^36,62,63^, amongst others^37,38^. With these current advancements in SMA therapeutics, biomarkers that are accurate, sensitive and widely available are required to monitor disease advancement or the efficacy of available and emerging therapeutics.

Extracellular vesicles, including microparticles and exosomes, are comprised of lipids, RNA and protein, the specific constituents of which is dependent on the cells from which they are derived. Work by other groups have identified exosomes as useful biomarkers for neurological conditions such as Creutzfeldt-Jakob disease^51^, and Parkinson’s disease^64^, as well as prostate cancer^57^, ovarian cancer^56^, cardiac injury^65^, kidney damage^58^, and many more. We have shown that SMN protein is released from cells in both microparticles and exosomes (Fig. 2). Moreover, the quantity of SMN protein within exosomes is reflective of the level expressed within the cell from which they are derived (Figs 3 and 4). Exosomes isolated from mouse and human SMA cell culture models demonstrated significantly lower SMN protein compared to controls (Fig. 4). Interestingly, our data also indicates that there is an increase in the level of exosomes in medium from cells that have reduced levels of SMN protein (Fig. 5). We also observed enhanced levels of circulating exosomes in serum samples from a mouse model of SMA and a human patient affected by SMA (Figs 6 and 7). Finally, we showed that SMN protein could be readily detected in exosomes isolated from mouse or human serum (Figs 6 and 7), and that the quantity of SMN protein contained in the serum-derived exosomes was reflective of the genotype, or disease state, in a mouse model (Fig. 6) and in patients (Fig. 7) with SMA. Taken together, our results suggest that SMN protein content in exosomes, or the quantity of exosomes contained in the serum itself, may represent a novel biomarker for SMA.

Previous efforts to develop blood-based biomarkers for SMA were largely unsuccessful. Several researchers have analyzed PBMCs for a variety of disease markers, including total SMN transcript level, relative full-length *versus* SMNΔ7 messenger RNA transcript level, and protein level^42,43,66–69^. Although several of these studies demonstrated a trend between motor function and several markers, no statistical correlation was observed. PBMC-based approaches can be significantly influenced by such things as underlying systemic infections that can alter SMN protein levels, likely due to the change in composition of immune cells circulating through the body^68,69^. Exosomes may offer a more precise, individualized and inexpensive tool for monitoring a patient’s disease state. Our data showing low quantities of SMN protein in serum-derived exosomes isolated from a mouse model of SMA, with carrier and wildtype mice showing correspondingly higher levels of the protein, suggest that this may serve as a useful biomarker for disease status in SMA.

Interestingly, using both cell culture and an animal model of SMA, in addition to preliminary work using patient samples, our work showed that SMN protein deficiency leads to impairment in exosome regulation, and enhanced levels of exosomes in the surrounding milieu relative to cells and mice expressing normal amounts of SMN protein (Figs 4–7). This finding will need to be reproduced in a larger cohort of patients with SMA. The mechanism behind this phenomenon is unclear. Reduced levels of SMN protein have been shown to reduce the release of synaptic vesicles^70^, alter intracellular vesicle trafficking^28^ and, more recently, endocytosis^71,72^. These deficiencies may be due to the impact of SMN proteins on actin dynamics within the cell^27,30,53^. Enhanced release of exosomes has been observed in other neurological disorders^73,74^ and cancer^75,76^. Enhanced release of exosomes may be an attempt by the cell to “correct” the microenvironment inside the cell, and expel toxic or unwanted factors. Recent work in a mouse model of SMA has suggested that mitochondrial dysfunction occurs during the presymptomatic stage of the disease leading to heightened levels of oxidative stress^77^, and oxidative stress can lead to enhanced release of exosomes in some cell types^78,79^. More recently, neurons from *Caenorhabditis elegans* were shown to release large (~4 μm) membrane-bound vesicles called exophers containing misfolded protein and organelles, and stressed cells that release these factors survive better than those in which release is blocked^80^. These observations suggest that one function of exosomes and other extracellular vesicles may be to actively clear unwanted elements from the cell, to improve cell health. Alternatively, given the recent studies showing that cells depleted of SMN protein have decreased endocytosis^71,72^, the increased levels of circulating exosomes could also be the result of impaired uptake, leading to their accumulation. Regardless, enhanced levels of extracellular vesicles from diseased tissues and cells in SMA tissue culture and animal models, and possibly in patients with SMA, may represent another viable biomarker of the disease state.

With the field of exosome biology consistently expanding, and novel findings on SMA pathology continuously arising, more research into how the SMA disease state alters exosome cargo, function and secretion is warranted. Future research should examine RNA and protein content within exosomes derived from both healthy and SMN-deficient cells to help elucidate potential role(s) for exosome secretion in the SMA disease state. Moreover, several groups have shown that exosome content can be manipulated for therapeutic purposes^81–83^, and therefore application of therapeutic exosomes may offer a unique strategy to deliver splicing modifiers or full length SMN protein to cells. Using two different approaches, we have shown that the quantity of SMN protein contained in exosomes can be dramatically enhanced (Fig. 3), providing a relatively simple way to generate “therapeutic” exosomes that may be able to deliver the protein to diseased cells in patients.

In conclusion, we have shown that microparticles and exosomes released from a variety of cell types contain full length SMN protein. In particular, exosomes display SMN protein levels that are reflective of their parental cell or animal’s disease state. Furthermore, cell culture and animal models of SMA demonstrate enhanced levels of exosomes in medium and serum, respectively, in comparison to their healthy controls. Taken together, these results suggest that serum-derived exosomes from patients may offer a novel biomarker for SMA.

## Methods

### Cell culture

293^84,85^, A549 (ATCC CCL 185^86^, and HeLa (ATCC CCL 2^87^) cells were grown in Minimum Essential Medium (MEM, Sigma, Oakville, ON) supplemented with 10% fetal bovine serum (FBS, Sigma), 2 mM GlutaMAX (Invitrogen, Burlington, ON), and 1X antimycotic-antibiotic (Invitrogen, Burlington, ON). HepG2 (ATCC HB-8065, ref.^88^), MN1^89^, and C2C12 cells^90^ cells have been previously described, and were grown in Dulbecco’s Modified Eagle Medium (DMEM, Sigma) supplemented with 10% FBS, 2 mM GlutaMAX and 1X antimycotic-antibiotic. Mouse embryonic fibroblasts (MEFs) derived from wildtype (MEF^WT^) or the *Smn* ^*2B*/−^ mouse strain (MEF^2B/−^) were isolated as described previously^53^, and were grown in DMEM supplemented as described above. An MN1-derived cell line with a stable knockdown of the *Smn* gene has been previously described^91^, and was kindly provided by Dr. Jocelyn Cote (University of Ottawa). Control normal human patient fibroblasts (GMO8333), SMA carrier fibroblasts (GMO3814) and SMA Type 1 fibroblasts (GMO3813) were obtained from Coriell Cell Repository, and were also grown in DMEM supplemented as described above. All cell lines were cultured at 37 °C and 5% CO_2_.

A549::SMN cells stably express an N-terminal FLAG-epitope tagged SMN protein, and was constructed as follows. pRP3129 contains the human cytomegalovirus (CMV) immediate early enhancer/promoter driving expression of a FLAG-tagged human *SMN1* cDNA linked to the hygromycin resistance gene through an internal ribosome entry site (IRES). A549 cells were transfected with pRP3129 using the Amaxa Cell Line Nucleofector Kit T (Lonza, Mississauga, Ontario), and pooled resistant cells were selected at 150 μg/ml hygromycin.

AdSMN is an early region 1 (E1) and E3-deleted vector containing an expression cassette comprised of the CMV immediate early enhancer/promoter driving expression of a FLAG-tagged *SMN1* cDNA and bovine growth hormone polyadenylation signal, and has been described previously^92,93^. AdSMN was propagated, purified and titered as previously described^94^. A549 cells were infected for 1 h at the indicated multiplicity of infection (MOI), and washed with phosphate buffered saline (PBS) immediately following infection to remove unattached virus. Infected cells were incubated in MEM lacking FBS for 24 h, and the media collected for exosome isolation as described below.

### Trichloroacetic acid media precipitation

Cells were seeded on 35 mm plates and, 24 h later, 1 ml of conditioned media was collected in a microfuge tube and centrifuged at 16,000 × g for 5 min. The cleared supernatant was added to 250 μl of >99% trichloroacetic acid **(**TCA, Sigma, Oakville, Ontario), and incubated on ice for 1 h. The solution was centrifuged at 16,000 × g for 5 min and the supernatant was discarded. The pellet was washed with 200 µL of 100% ethanol and centrifuged at 16,000 × g for 5 min. The resulting pellet was resuspended in 100 µL 2x protein loading dye (62.5 mM Tris-HCl pH 6.8, 25% glycerol, 2% SDS, 0.01% bromophenol blue and 5% β-mercaptoethanol). Samples were stored at −20 °C until the time of analysis.

### Isolation of microparticles and exosomes

Extracellular vesicles were isolated from the medium of cultured cells at the indicated time points using one of two protocols. The method of exosome isolation for each experiment is indicated in the figure legend and in the text of the manuscript. Extracellular vesicles were typically isolated from cells grown for the indicated time in media lacking FBS or supplemented with microvesicle-depleted FBS, as indicated in the figure legend. For isolation of extracellular vesicles using differential centrifugation, conditioned medium was initially centrifuged for 10 min at 500 × g to remove cells and large debris. The resulting supernatant was subsequently centrifuged at 2,500 × g for 10 min to obtain a pellet of apoptotic bodies, which was resuspended in PBS or RIPA buffer (150 mM NaCl, 1.0% NP-40, 0.5% sodium deoxycholate, 0.1% sodium dodecyl sulfate, 50 mM Tris, pH 8.0). Microparticles were then pelleted from the supernatant through centrifugation at 20,000 × g for 20 min at 4 °C, and resuspended in PBS or RIPA buffer. Finally, exosomes were isolated using a 100,000 × g centrifugation for 90 min at 4 °C, and the resulting pellet was resuspended in PBS or RIPA buffer.

Alternatively, exosomes were isolated using the commercially available Exoquick kit (System Biosciences, Mountain View, CA, EXOQ5A-1 for serum samples, EXOTC50A-1 for tissue culture media samples) according to the manufacturer’s instructions. For all methods of isolation, the final pellet was resuspended in 100 µL of PBS or RIPA. Exosome protein concentration was determined using the Bradford assay (BioRad, Hercules, CA). Exosome size and particle concentration was determined using the Zetaview PMX110 Multiple Parameter Particle Tracking Analysis (ParticleMetrix, Meerbusch, Germany) in size mode. Vesicles were resuspended in 1x PBS and diluted to the working range of the system (10^6^–10^9^ particle/ml). Videos were captured and analyzed with the ZetaView software (version 8.02.28, Meerbusch, Germany) using 11 camera positions, a 2-second video length, and a camera frame rate of 15 fps (for microparticles) or 30 fps (for exosomes) at 21 °C.

### Immunoblot analysis

Protein samples were isolated either directly in 2x protein loading dye, or PBS or RIPA buffer when protein quantification was required. For the latter samples, following Bradford protein quantification assay, the desired amount of protein was diluted in 2x protein loading buffer. Samples were heated for 5 min at 95 °C, separated by 9 or 15% SDS-PAGE and transferred to a polyvinylidene difluoride membrane (Millipore). The membrane was blocked in 5% milk and probed with the following antibodies: rabbit anti-Alix (Sigma, Oakville, Ontario) at 1:1,000, mouse anti-Flag (Sigma, Oakville, Ontario) at 1:10,000 for cell lysates or 1:1,000 for exosomes, rabbit anti-flotillin-2 (Cell Signaling, MA) at 1:1,000, rabbit anti-Tsg101 (Cell Signaling, MA) at 1:1,000, mouse anti-SMN (BD Transduction, Mississauga, Ontario) or rabbit anti-SMN (Santa Cruz, Dallas TX) at 1:10,000 for cell lysates or 1:1,000 for exosomes, or mouse anti-Tubulin (EMD Millipore, Etobicoke, Ontario) at 1:10,000. Binding of the primary antibody was detected using a goat anti-mouse (BioRad, Hercules, CA) at 1:10,000, rabbit anti-goat (Sigma) at 1:10,000 or mouse anti-rabbit conformation specific (Cell Signaling, MA) at 1:1,000 secondary antibody conjugated to horseradish peroxidase, and visualized by chemiluminescent reaction (Pierce, Thermo Scientific). Signal intensities were quantified using Image J (version 1.51, developed by Wayne Rasband, NIH) or Image Studio Light (Licor, Lincoln, Nebraska), and normalized to the appropriate loading control. Scanned images of immunoblots were cropped to remove excess image and border.

### Immunogold labeling for transmission electron microscopy

Exosomes were isolated from 12 ml of conditioned medium from A549 cells using the Exoquick method. The exosome pellet was fixed for 1 h at room temperature (RT) in 500 μl of Karnovsky’s fixative (4% paraformaldehyde, 2% glutaraldehyde and 0.1 M sodium cacodylate in phosphate-buffered saline, pH 7.4). Exosomes were centrifuged at 20,000 × g for 1 min and the supernatant removed. After fixation, the exosome pellet was subsequently washed 3 × 10 min in 0.1 M sodium cacodylate buffer. The fixed exosomes were permeabilized through incubation for 10 min in 500 μl of 0.1% Triton X-100 in 0.1 M sodium cacodylate buffer. The exosome pellet was incubated in 500 μl of blocking buffer (5% goat serum in 0.1 M sodium cacodylate buffer and 0.05% Triton x-100) for 30 min at RT. After centrifugation, the exosome pellet was incubated for 2 h at RT with primary antibody (purified mouse anti-SMN, BD Transduction Laboratories) diluted 1:10–1:20 in blocking buffer. Following the incubation period, the pellet was washed with 500 μl of blocking buffer 3 × 10 min. The exosome pellet was incubated at RT with a goat polyclonal antibody to mouse IgG conjugated with 10 nm gold (Abcam) diluted 1:40–1:50 in blocking buffer for 1 h in the dark. Samples were washed with 500 μl of 0.1 M sodium cacodylate buffer 3 × 10 min. Immunogold-labelled exosomes were fixed with 2% glutaraldehyde in 0.1 M sodium cacodylate buffer overnight, then washed with 0.1 M sodium cacodylate buffer 4 × 10 min. The exosomes were post-fixed with 1% osmium tetroxide in 0.1 M sodium cacodylate buffer for 60 min and washed in distilled water 3 × 5 min. Specimens were dehydrated in an ascending concentration of alcohol in water (30-50-70-85-95%) using molecular sieve type 3 A (EMD) for 10 min each step followed by 10 min incubation in 100% ethanol. Exosomes were washed 10 min in 50% ethanol/50% acetone and then in 100% acetone for 10 min. Exosomes were incubated in 30% Spurr resin/acetone overnight (~15 h), 50% Spurr resin/acetone for 6 h, and in fresh 100% Spurr resin overnight on a rotating platform. Spurr resin was changed 3 times over the next 24 h period. The immunogold-labeled exosomes were finally embedded in fresh liquid Spurr resin, which was polymerized overnight at 70 °C. The specimen embedded in the resin was cut with an ultramicrotome (80 nm sections). Ultrathin sections of exosomes were collected onto 200-mesh copper grids and stained with 2% aqueous uranyl acetate and with Reynold’s lead citrate. These sections stained on grids were observed under a transmission electron microscope (Hitachi 7100) at 100,000x and 150,000x magnifications.

### Animal Studies

The *Smn* ^*2B/2B*^ mouse strain has been previously described^30,53,95,96^. This mouse strain has a knock-in allele containing a 3 nucleotide substitution within the exon splice enhancer (ESE) of exon 7 of the mouse *Smn* gene, resulting in alternative splicing of the mouse *Smn* transcript and preferential production of *Smn* mRNA lacking exon 7. These mice were maintained on a C57Bl6/J background, and were bred and genotyped to identify affected *Smn* ^*2B*/−^, carrier *Smn* ^*2B/+*^, and wildtype littermates, as previously described^97^. Experimental animals were cared for in the Animal Care Facility at the University of Ottawa, and kept in a conventional animal house with a constant room temperature of 24 °C and a 12 h dark/light cycle, and with free access to food and water. Mice were monitored daily by animal facility professionals. Post-natal day 18 (P18) pups were anesthetized with isofluorane, blood collected by cardiac puncture, and processed with Exoquick (System Biosciences, EXOQ5A-1) to isolate exosomes from serum. Samples were stored at −80 °C until analysed.

### Patient Serum Samples

A 29 year old male patient with SMA Type 3 (homozygous deletion of *SMN1*), and an age-matched male healthy control, gave informed consent to participate in the Care4Rare Canada research study, which was approved by the Children’s Hospital of Eastern Ontario and Ottawa Health Science Network Research Ethics Board (OHSN-REB). Human blood was collected in BD red cap Vacutainer tubes, inverted several times, and left to coagulate for 30–45 min at RT. The clot was then removed by centrifugation at 1000 × g for 10 min at 4 °C. Serum was aliquoted into tubes and stored at −20 or −80 °C. Exosome isolation continued with the Exoquick reagent (System Biosciences, Mountain View, CA, cat#EXOQ5A-1) as described above, and analyzed for exosome size and concentration using the ZetaView system or immunoblot as described above.

### Statistical Analysis

Statistical analyses were performed using IBM SPSS Statistics 21. Paired/unpaired T-tests, ANOVA and regression analysis were completed with significance set to p < 0.05. Levene’s test for variance was performed to determine the appropriate post-hoc analysis. Post-hoc analysis was performed using the Bonferroni t-test if there was homogeneity of variance, while other cases utilized Games Howell’s post hoc test.

### Data Availability Statement

All data generated or analysed during this study are included in this published article.

### Study Approvals

All animal experiments were approved by and performed according to the guidelines set by the Animal Research Ethics Board at the University of Ottawa (Ottawa, ON, Canada) (Protocol Number OHRI2152). Human subjects gave informed consent to participate through the Care4Rare Canada research study, which was approved by the Children’s Hospital of Eastern Ontario and Ottawa Health Science Network Research Ethics Board (OHSN-REB, Protocol Number 20150232-01H). All methods were performed in accordance with the relevant guidelines and regulations of the OHSN-REB.

## Electronic supplementary material

Supplemental Data
