# Supplementary material for: Survival Motor Neuron Protein is Released from Cells in Exosomes: A Potential Biomarker for Spinal Muscular Atrophy
Source: Sci Rep. 2017 Oct 24;7:13859. doi: 10.1038/s41598-017-14313-z (PMC5655039; doi:10.1038/s41598-017-14313-z)
Supplement: Supplementary file 1 — Supplemental Data [file 41598_2017_14313_MOESM1_ESM.pdf]

Survival Motor Neuron Protein is Released from Cells in Exosomes: A Potential Biomarker for Spinal Muscular Atrophy

Leslie A. Nash[1-3], Emily R. McFall[1-3], Amanda M. Perozzo[1], Maddison Turner[4,5], Kathy L. Poulin[1], Yves De Repentigny[1], Joseph K. Burns[1-3], Hugh J. McMillan[3,6,7], Jodi Warman Chardon[3,5,7-9], Dylan Burger[4,5], Rashmi Kothary[1,3,5,9], and Robin J. Parks[1-3,9,10]

[1]Regenerative Medicine Program, Ottawa Hospital Research Institute, Ottawa, Ontario, Canada,
[2]Department of Biochemistry, Microbiology and Immunology, University of Ottawa, Ottawa, Ontario, Canada,
[3]University of Ottawa Centre for Neuromuscular Disease, Ottawa, Ontario, Canada,
[4]Kidney Research Centre, Ottawa Hospital Research Institute, Ottawa, Ontario, Canada,
[5]Department of Cellular and Molecular Medicine, University of Ottawa, Ottawa, Ontario, Canada,
[6]Department of Pediatrics, Children's Hospital of Eastern Ontario, University of Ottawa, Ottawa, Canada,
[7]Children's Hospital of Eastern Ontario Research Institute, Ottawa Ontario, Canada,
[8]Division of Neurogenetics, Department of Genetics, Children's Hospital of Eastern Ontario, Ottawa, Canada,
[9]Department of Medicine, University of Ottawa, Ottawa, Ontario, Canada

Supplementary Figure S1

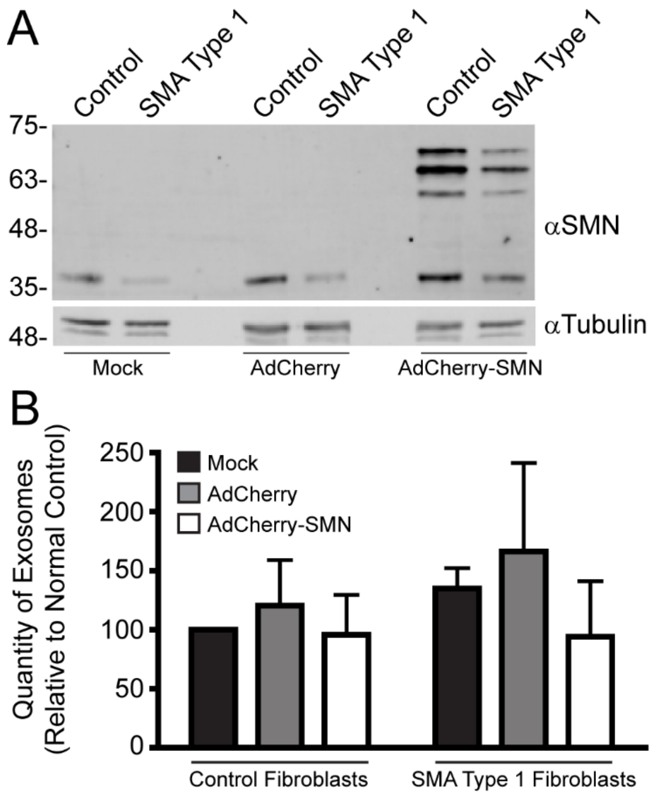

Supplementary Figure S1. Analysis of exosome release after restoration of SMN protein levels in fibroblasts from a patient with type 1 SMA. Control fibroblasts or cells from a patient with SMA type 1 were plated at a density of $1.1x10^6$ cells per dish in 10 cm plates. Twenty-four hr later, the cells were infected at a multiplicity of infection of 250 for 3 hr with an E1/E3-deleted adenovirus vectors expressing the mCherry reporter protein (AdCherry) or an mCherry-SMN fusion protein (AdCherry-SMN), or left uninfected (mock). After infection, the virus inoculum was removed, the cells were washed three times with PBS, and the culture medium replaced with medium containing exosome-depleted fetal bovine serum. Seventy-two hr later, media was collected from the cells, and exosomes were isolated using Exoquick reagent, according to the manufacturer's instructions. The cells were also collected for analysis of protein expression by immunoblot. Additional details on the specific materials and methods are as described in the main text of the manuscript. Panel A: Crude protein extracts from the treated cells were separated by SDS-PAGE, and subjected to immunoblot for SMN protein or tubulin (loading control). Panel B: The concentration of exosomes in the medium was determined using the Zetaview ParticleMetrix system, and is reported as quantity of exosomes in the medium relative to the mock-treated control fibroblasts, and shown as average and standard deviation (n=3). Analysis of the data by paired t-test did not yield statistically significant differences between the treatments.
